# Books Reviewed

**Published:** 1866-10

**Authors:** 


### Books Reviewed.

Da Costa's Medical Diagnosis. Second edition.

It is but a few months since the first edition of this work was placed upon our table and noticed in our pages. We have but little to add, to what we said on the former occasion. The sale of a very large edition in the short space of eighteen months shows conclusively that the profession agree with us in our opinions of the work, and that it has been received with great favor. The author says in his preface to his second edition that "the volume has been revised, and about ninety pages and twenty-two wood-cuts added, mainly on subjects which in the former edition were but briefly touched upon. The chief additions will be found in the chapters on Diseases of the Brain, of the Larnyx, of the Blood on the Urine, and on Paracites, and in the section on Abdominal Enlargement, though new matter has also been incorporated in other parts of the book." This work as a guide in the discrimination of disease, is without doubt unexcelled in our language, and owes its deserved popularity to its clear and truthful descriptions of disease as well as the discriminations made between different diseases.

A careful analysis of the symptoms of disease is indispensable, in correct diagnosis, and the daily habit of investigation and comparison is necessary to appreciate their import and the relation they hold to each other. The elements of a correct and accurate diagnosis are happily presented in this book, and its careful pursual will repay the attentive reader. Mental acumen, the power of nice discrimination, the ability to judge correctly are essential for correct diagnosis, not less than faithful descriptions of disease and knowledge of the symptoms by which diseases are manifested. It is perhaps in this respect more than in any other, that mental strength is manifested, and nothing adds so much to the reputation and standing of a physician as the ability to discriminate correctly the import and relation of the various symptoms of disease. The general plan of this work, and the numerous subjects of which it treats have been already described and are sufficiently understood by all our readers. This volume will be highly prized by all who would understand the nature of disease and accurately discriminate the import of its varying symptoms.

---

Transactions of the Indiana State Medical Society at its Sixteenth Annual Session, held at Indianapolis, May 15th, 16th and 17th, 1866. Published for the Society.

This volume of the Transactions of the Indiana State Medical Society has several interesting and valuable essays embodied within its pages. The President, Dr. Harding, in his Inaugural Address, directs the attention of the Society to the effects of climate and temperature upon the health and national character of communities, and says that "much that is obscure and imperfectly understood, as yet, of the causation of disease, and especially of an endemic or epidemic character, will be connected and explained by a more familiar acquaintance with laws governing climatic and meteorological phenomena. This broad and comparatively uncultivated domain belongs, legitimately, to the medical profession; and doubtless some patient and philosophic laborer in this important field of inquiry, will yet make discoveries, as to the cause and prevention of disease, that will shed lustre on our profession, and entitle the discoverer to the distinction of being classed with the greatest benefactors of the human family."

---

Medical Electricity; Nervous Diseases. By ALFRED C. GARRATT, M. D., Fellow of the Massachusetts Medical Society; Member of the American Medical Association, etc., etc.

This is a volume of near 1100 pages, and must contain all that is known of Electricity and Electro-Therapeutics. We have not had time to carefully peruse the book, and must confess personal ignorance of nearly the whole subject. Electricity probably has some influence in disease, but we have never been able to make it available as a remedy, and have consequently no very great confidence in its curative virtues. Without expressing any very positive opinions or claiming much experience we propose to give our readers a quotation from the author's preface, hoping in this way to give a more truthful idea of the work than could be otherwise possible.

"When I first directed my entire professional labors to this difficult department of *special* medical practice, (after having been engaged in the general practice of medicine for nearly twenty years,) to speak mainly in the words of another—I did so with the fullest sense of their importance, in two relations: first, as they related to my own future career and reputation; second, as related to the advancement of the healing art, and the immediate relief of a no small class of otherwise unreached, afflicted, and suffering persons. I was fully aware that my position, my views, and aims might excite misapprehension, because the hitherto very general association of the empirical uses of electricity, with quackery, throughout the length and breadth of our country, would naturally lead to some erroneous verdict, at least until my true position might be correctly and definitely defined.

But one word further. Our art is *one* art. Each branch is but a part of the whole, and simply, "*e pluribus unum.*" It is too late to be sticklers for creeds or isms, for pathies or systems; only let each be honest and earnest in his professional sphere. The author is desirous that this should no longer be termed a "*System*" of practice, but merely *the electric remedies*, etc., and that we take special pains to eradicate those false notions from the minds of the people.

I wish here to call particular attention to the fact, that almost no allusion is made in this work to the simultaneous employment of medicines with electric treatment. This is *purposely* omitted; but it must not necessarily follow that it is to be omitted in practice, if we wish to gain the greatest possible amount of improvement for the patient in the least possible time. Indeed, it will often be noticed that a skillful use of electric carrents will *quicken* the action, and *heighten* the effects of internal medicines. Often, cases will be presented that promise success only in this way.

Again: like many other potent remedies now, as heretofore, employed in the treatment of diseases, electricity is greatly valued for its given effects, in certain cases, by a small portion of the profession who have thoroughly investigated it, among whom are some of the most distinguished names; while another portion of tho profession, equally respectable, think but little of it; and others there are who discard it altogether. But when it is shown that the nerves, muscles, and many of the secretions can be more surely and more uniformly called into their natural action by means of electricity than by any other known agent, and that the *degree* and *kind* of that effect is *widely different*, according to the form, quantity, or intensity of the electricity employed, and that again modified *as widely*, according to the *methods* of administering the dose at each *séance*, it is to be expected that the existing differences of opinions as to the healing power, or the manageable and remedial value of electricity, will be more nearly harmonized, and that on an intelligent basis.

It must not be thought certain that the electric current exercises an inworking influence only on or through the nerves and muscles. It is, on the contrary, my intention to aid medical men to become familiar with the idea, that all textures of the living animal body, being saturated, as they are, with the saline solutions of the blood and other secretions of the animal economy, are peculiarly accessible to the chemical and mechanical workings of the static, galvanic, and electro-magnetic currents. However, to my own mind, it is only by the most minute and slowly maturing experience in the analagous workings of these currents in different living tissues, that insight and confidence can be obtained, which are so necessary for the rational application of electric currents for curing diseases. I trust this work will present a phalanx of facts, as well as many original, practical directions for obtaining physiological and therapeutical results, that will be found worthy of a candid attention, and lead on to fresh researches in this inviting department of medical science.

Finally, the author of this work has aimed by directness, thoroughness, and extent of practical research, thus presented by himself or by accredited authorities; by ample plates of apparatus, and of anatomy; by great simplicity in style, and freedom from technicalities as far as possible, (also by *term* Explanations,) to present this whole subject of *Medical Electricity* in so clear and simple a manner as to be readily understood by any one of ordinary intelligence; hoping it may invite into this hitherto neglected, but intensely interesting and profitable study of Electricity, as it relates to human life and health, *to the cause and cure of disease*, all ranks of the medical profession, as well as help to initiate the younger candidates for its labors and its honors, in years to come, to a still more *rational view* of diseases and their remedies; to all of whom, or whosoever reads, it may prove an exposition of this subject at once elementary, practical and substantial."

Possibly our readers can appreciate from the author's own description of his book something of what he has attempted. The work is more obnoxious to criticism than most books upon scientific subjects; but, perhaps, the earnest seeker for truth may find it within the compass of this volume.

---

Instructions on the Preparation, Administration and Properties of Nitrous Oxide, Protoxide of Nitrogen or Laughing Gas. By GEORGE F. BARKER, D. D. S., Professor of the Principles of Dental Surgery and Therapeutics in the Philadelphia College of Dental Surgery, etc., etc. Philadelphia: Rubencame & Stockton, 1866.

The peculiar exhilarating properties of nitrous oxide have long been known, and its employment as an internal remedy has been repeatedly practiced for some time however with but little success. The monograph under consideration is especially devoted to the discussion of its anæsthetic properties, attention to which has recently been called, the author claiming for it a prominent place in the list of anæsthetics, and ascribing to it properties possessed by no other similar agent with which we are acquainted. He presents the subject in a lucid and practical manner, considering in succession the properties both chemically and physiologically of nitrous oxide, its mode of its administration and the apparatuses employed for the same. An instrument for generating the gas has been invented by the author, by which he is enabled to keep it on hand for any length of time. We would cheerfully recommend to all those of the profession who desire to investigate this subject and those who employ this agent for its anæsthetic properties, this work, which embodies all that is known regarding its preparation and mode of administration.

